# Investigation on the Rationality of the Extant Ways of Scoring the Interpersonal Reactivity Index Based on Confirmatory Factor Analysis

**DOI:** 10.3389/fpsyg.2020.01086

**Published:** 2020-06-03

**Authors:** Yang Wang, Yun Li, Wanting Xiao, Yuanshu Fu, Jing Jie

**Affiliations:** ^1^School of Public Administration, Guangdong University of Finance, Guangzhou, China; ^2^School of Psychology, South China Normal University, Guangzhou, China; ^3^Guangzhou Rehabilitation and Research Center for Children with Autism, Guangzhou Cana School, Guangzhou, China; ^4^School of Education, Zhaoqing University, Zhaoqing, China; ^5^Center for Mental Health Education, Hainan University, Haikou, China

**Keywords:** empathy, Interpersonal Reactivity Index, scoring approaches, confirmatory factor analysis, model-based reliability

## Abstract

As the most frequently used tool for measuring empathy, the Interpersonal Reactivity Index (IRI) is often scored by researchers arbitrarily and casually. Many commonly used IRI scoring approaches and their corresponding measurement models are unverified, which may make the conclusions of subsequent variable relation studies controversial and even misleading. We make the first effort to summarize these measurement models and to evaluate rationality of the common scoring methods of the IRI by confirmatory factor analysis, focusing on model fitting, factor loading, and model-based reliability simultaneously. The results show that most of these models do not fit well, indicating that the scoring approaches of the IRI corresponding to these models may be problematic. Relatively speaking, better scoring approaches of the IRI include summing empathic concern (EC) and perspective taking (PT) as the total score of the IRI and reporting the score of PT as cognitive empathy.

## Introduction

Empathy is usually defined as the ability to experience and understand the emotions of others, which plays a very important role in people’s social life and emotional communication (Villadangos et al., 2016). It has a great influence on the psychology and behavior of both the empathizers and their target persons. Research on empathy has been a hot topic in the field of psychology for a long time, which is largely due to the continuous development of the psychometric tools of empathy, especially self-report measures. So far, dozens of self-report measures of empathy have been developed. Among them, the most common measure is the Interpersonal Reactivity Index (IRI; Davis, 1983). The IRI is a multidimensional measure that consists of four subscales: perspective taking (PT) measures the ability to shift to another person’s perspective, empathic concern (EC) measures other-oriented feelings of sympathy and concern for others, fantasy (FS) measures the tendency to become imaginatively absorbed in the feelings and actions of characters in books and movies, and personal distress (PD) measures self-oriented feelings of personal anxiety and uneasiness in tense interpersonal settings.

Although the IRI is developed as a four-dimension scale, researchers often use the IRI scores flexibly in their studies based on the different definitions and constructs of empathy that they advocate. For example, researchers who regard empathy as a general construct always add up the four dimensions of the IRI to obtain a total empathy score (e.g., Sun et al., 2018) or add EC and PT together as the overall empathy score (e.g., Nicol and Rounding, 2013). Some researchers abandon the PD dimension because of its low or even negative correlation with the other dimensions and add PT, EC, and FS to represent empathy (e.g., Pulos et al., 2004). In addition, researchers who recognize empathy as a dualistic construct combine the EC and PD subscales into an “affective empathy” factor and combine the PT and FS subscales into a “cognitive empathy” factor of the IRI (e.g., Fan and Hu, 2017). Some researchers also combine EC and PD to represent affective empathy, but only PT is retained as cognitive empathy (e.g., He and Zhu, 2016). Some others only regard EC and PT as representatives of affective empathy and cognitive empathy, respectively (e.g., Luo et al., 2013). Additionally, some researchers consider interpersonal reactivity as a higher order factor and regard empathy (measured by EC and PT), FS, and PD as its dimensions (e.g., Siu and Shek, 2005).

To conclude the application of the IRI mentioned above, we find that researchers have used the IRI scores in several ways based on different measurement models, but most of these models and scoring ways have not been verified. At present, the psychometric analysis of the IRI is mainly verifying its original four-factor structure (even if the four-factor model is adopted, many psychometric studies fail to provide model-fitting information, e.g., Zhang et al., 2010; Guan and Qian, 2014). However, even though the four-factor model of the IRI fits well and can be used reasonably, it does not mean that other models (such as the three-factor model with only PT, EC, and FS representing empathy) are equally valid.

To address this concern, some researchers have tried to verify other commonly used measurement models other than the above four-factor model in recent years. Chrysikou and Thompson (2016) found that the two-factor model that treats EC and PD as affective empathy and FS and PT as cognitive empathy was poorly fitted. Murphy et al.’s (2020) study is another example that found the three-factor model of interpersonal reactivity did not fit well. However, some problems remain unresolved. First, the aforementioned two studies only focus on a limited number of measurement models of the IRI, and several other measurement models mentioned above still have not been verified, some of which are frequently used in empirical studies. For example, many studies use EC and PT to represent affective empathy and cognitive empathy, respectively, and add them to reflect general empathy. This way of scoring has a precondition: a high-order or bi-factor model containing both local factors of EC and PT is constructed first, and the model is at least confirmed to fit well. Unfortunately, this precondition has yet to be examined. The above scoring method is justified solely by reporting the respective and total alpha coefficients of the two dimensions. Second, both Chrysikou and Thompson (2016) and Murphy et al. (2020) mainly focus on the model-fitting problem and fail to uncover more information from confirmatory factor analysis, such as the model-based reliability index (e.g., homogeneity reliability, composite reliability, and residual reliability); it can tell us directly which dimensions and the combined scores of the dimensions make sense from a statistical perspective. We should know that, even if the model fits well, it is unreasonable to use dimension scores or total scores while their model-based reliability indexes are unacceptable (Ye and Wen, 2012).

In summary, this study aims to set up and verify a series of IRI measurement models by confirmatory factor analysis and calculate the model-based reliability to provide direct psychometric evidence for the reasonable use of various IRI scorings in existing studies. This issue is of great significance to empathic empirical research because IRI is undoubtedly the most frequently used psychometric tool for empathy research. If researchers use the IRI scores incorrectly, the subsequent study results and conclusions may be misleading. Therefore, it is necessary for researchers to know which scoring approaches are feasible and which are not. Our study is the first effort to directly evaluate the rationality of the common scoring methods of the IRI.

## Materials and Methods

### Participants

The research samples were collected from six universities in the provinces of Guangdong (3), Gansu (1), and Jiangxi (2) in mainland China. Using a convenience sampling approach and an online survey, researchers from each university were entrusted to provide the web links to the questionnaire. All subjects were informed of the purpose of the research in advance; additionally, they were assured that their participation would be completely anonymous and that their responses would be kept strictly confidential and only be used for academic purposes. After the completion of the questionnaire, 533 valid questionnaires were recovered automatically by the questionnaire system. Among them, 204 were male, and 329 were female; 238 participants were majoring in arts, and 295 were majoring in science. With regard to education level, there were 256 undergraduate students, 108 postgraduate students, five doctoral students, and 28 students with no information provided. The average age was 20.79 ± 2.89 years, and 16 people did not provide information on age.

### Measures

#### Interpersonal Reactivity Index (IRI)

The Chinese version of the IRI (IRI-C; Zhang et al., 2010) consists of 22 items with four dimensions: PT (e.g., “I try to look at everybody’s side of a disagreement before I make a decision”), EC (e.g., “I often have tender, concerned feelings for people less fortunate than me”), FS (e.g., “I really get involved with the feelings of the characters in a novel”), and PD (e.g., “In emergency situations, I feel apprehensive and ill-at-ease”). Every item is rated on a five-point Likert scale ranging from 1 (does not describe me well) to 5 (describes me very well).

#### Prosocial Tendencies Measure (PTM)

The Chinese version of the Prosocial Tendencies Measure (PTM) (Kou et al., 2007) consists of 26 items (e.g., “I can help others best when people are watching me”). Participants rated the items on a five-point Likert scale ranging from 1 (“Does not describe me well”) to 5 (“Describes me greatly”). In the current study, the homogeneity coefficient was 0.78 for the PTM.

#### Buss-Perry Aggression Questionnaire (BPAQ)

The Chinese version of the Buss-Perry Aggression Questionnaire (BPAQ; Lv et al., 2013) consists of 22 items (e.g., “Given enough provocation, I may hit another person”). Participants rated the items on a five-point Likert scale ranging from 1 (“Strongly disagree”) to 5 (“Strongly agree”). In the current study, the homogeneity coefficient was 0.73 for the BPAQ.

### Statistical Analysis

With an aim to use the IRI score from the previous studies mentioned above, the following measurement models were established and verified: a four-factor model, a four-factor high-order model, and a four-factor bi-factor model (hereafter M_1a_, M_1b_, M_1c_); a three-factor (PT, EC, and FS) model and its corresponding high-order factor model and bi-factor model (hereafter M_2a_, M_2b_, M_2c_); a three-factor model with empathy (EC+PT), FS, and PD as three independent factors of interpersonal reactivity and its corresponding high-order factor model and bi-factor model (hereafter M_3a_, M_3b_, M_3c_); a two-factor model with PT as the cognitive empathy factor and EC as the affective empathy factor and its corresponding high-order factor model and bi-factor model (hereafter M_4a_, M_4b_, M_4c_); a two-factor model with PT and FS as the cognitive empathy factor and EC and PD as the affective empathy factor and its corresponding high-order factor model and bi-factor model (hereafter M_5a_, M_5b_, M_5c_); and a two-factor model with PT as the cognitive empathy factor and EC and PD as the affective empathy factor and its corresponding high-order factor model and bi-factor model (hereafter M_6a_, M_6b_, M_6c_).

Seven traditional indexes, *χ*^2^/*df*, CFI, NNFI, SRMR, RMSEA, AIC, and BIC were used to evaluate the model fit. Based on the extensive methodological literature and the customary practice of empirical research, values not greater than 3 and 2 for *χ*^2^/*df*, respectively, support acceptable and good model fit; values higher than 0.90 and 0.95 for CFI and NNFI, respectively, support acceptable and good model fit; values not greater than 0.08 and 0.06 for RMSEA, respectively, support acceptable and good model fit; values not greater than 0.08 for SRMR support acceptable model fit; smaller AIC and BIC indicate a better model fit (Schermelleh-Engel et al., 2003; Hancock and Mueller, 2013; Finch and French, 2015; Xia and Yang, 2019). Traditional fit indexes have some limitations, such as most of them are merely descriptive. In other words, these indexes tell us neither the probability of errors when accepting a model, nor the possible size of misspecification. To provide this additional information on the model fit, we add a new index, RMSEA*_*t*_* based on equivalence testing (ET) to evaluate model. Values not greater than the empirical critical value (i.e., 0.08 or 0.06) for RMSEA*_*t*_* indicate that, if we accept the model, misspecification does not exceed RMSEA*_*t*_*, and the probability of committing a type I error is not more than 0.05. Readers interested in the basic knowledge of ET and RMSEA*_*t*_* can refer to Yuan et al. (2016).

For those models whose fit meets the recommended cutoff, three model-based reliability indexes were calculated. For a multidimensional scale consisting of *p* items (*x*_1_, *x*_2_,…, *x*_*p*_) and measuring a general factor *G* as well as several *n* local factors (*F*_1_, *F*_2_,…, *F*_*n*_), item *x*_*i*_ can be denoted as

(1) $${\text{x}}_{\text{i}}={\text{a}}_{\text{i}}\,G+\sum_{\text{j}=1}^{\text{n}}{\text{b}}_{\text{ij}}\,F_{\text{j}}+\delta_{\text{i}},i=1,2,\ldots \,p$$

where *a*_*i*_ and *b*_*ij*_ are *x*_*i*_’s loading on general factor *G* and local factor *F*_*j*_, respectively; *δ_*i*_* is *x*_*i*_’s measurement error. Global factors, local factors, and errors are usually assumed to be independent of each other. Then, homogeneity coefficient (aka, *ω_*H*_* or omegaH), which provides information about the extent to which total scores were interpretable as a single general factor, can be computed by the following formula:

(2) $$\omega_{H}=\frac{{\left(\sum_{i=1}^{p}a_{i}\right)}^{2}\,v\,a\,r\,\left(G\right)}{{\left(\sum_{i=1}^{p}a_{i}\right)}^{2}\,v\,a\,r\,\left(G\right)+v\,a\,r\,\left(\sum_{i=1}^{p}\sum_{j=1}^{n}b_{i\,j}\,F_{j}\right)+\sum_{i=1}^{p}v\,a\,r\,\left(\delta_{i}\right)}$$

A high enough *ω_*H*_* (e.g., >0.50; see Reise et al., 2013) means that it is meaningful to calculate the total score of all the dimensions (Rodriguez et al., 2016a, b). (2) Composite reliability of subscale (aka, *ω_*S*_* or omegaS) for dimension *j* can be computed by the following formula:

(3) $$\omega_{S_{\text{j}}}=\frac{{\left(\sum_{i=1}^{p_{\text{j}}}a_{i}\right)}^{2}\,v\,a\,r\,\left(G\right)+{\left(\sum_{i=1}^{p_{\text{j}}}b_{i\,j}\right)}^{2}\,v\,a\,r\,\left(F_{j}\right)}{{\left(\sum_{i=1}^{p_{\text{j}}}a_{i}\right)}^{2}\,v\,a\,r\,\left(G\right)+{\left(\sum_{i=1}^{p_{\text{j}}}b_{i\,j}\right)}^{2}\,v\,a\,r\,\left(F_{j}\right)+\sum_{i=1}^{p_{\text{j}}}v\,a\,r\,\left(\delta_{i}\right)}$$

(3) Residualized reliability (aka, *ω_*HS*_* or omegaHS) for dimension *j* can be computed by the following formula:

(4) $$\omega_{H\,S_{\text{j}}}=\frac{{\left(\sum_{i=1}^{p_{\text{j}}}b_{i\,j}\right)}^{2}\,v\,a\,r\,\left(F_{j}\right)}{{\left(\sum_{i=1}^{p_{\text{j}}}a_{i}\right)}^{2}\,v\,a\,r\,\left(G\right)+{\left(\sum_{i=1}^{p_{\text{j}}}b_{i\,j}\right)}^{2}\,v\,a\,r\,\left(F_{j}\right)+\sum_{i=1}^{p_{\text{j}}}v\,a\,r\,\left(\delta_{i}\right)}$$

*ω_*S*_* and *ω_*HS*_*, respectively, provide information about the variability of subscales before and after controlling the variance due to the general factor. A high enough *ω_*S*_* and a high enough ratio between *ω_*HS*_* and *ω_*S*_* (hereafter *ω_*HS*_*/*ω_*S*_*) (e.g., >0.50; see Reise et al., 2013) mean that it is meaningful to report the score of the subscale (Rodriguez et al., 2016a; Gu and Wen, 2017). Additionally, an item explaining common variance (I-ECV) for each well-fitted model was computed by dividing the squared item loadings on the general factor by the sum of squared item loadings on the general factor and local factor. Items with a high I-ECV (e.g., >0.80, see Rodriguez et al., 2016b) can be considered strong indicators for the general factor relative to local factor.

After the above analysis, we conducted a measurement invariance analysis across gender to further evaluate the internal structure of the best-fitted model. Referring to the previous research on measurement invariance of the IRI (e.g., Lucas-Molina et al., 2017), we successively tested four levels of group invariance, including configural, metric (i.e., factor loading invariance), scalar (i.e., intercept invariance), and latent mean invariance. According to the recent simulation and empirical research, the traditional fit indexes of measurement invariance, such as Δ*χ^2^* and ΔCFI, are unable to control either type I or type II errors (Yuan and Chan, 2016; Finch and French, 2018). The statistical properties of the fit based on ET (e.g., RMSEA*_*t*_*) are obviously superior to traditional indexes (Counsell et al., 2019). Therefore, RMSEA*_*t*_* is used as the fit index in invariance analysis. For the model with additional parameter restrictions, an RMSEA*_*t*_* not greater than the empirical cutoff indicates that, if we accept the model, misspecification caused by parameter restrictions does not exceed RMSEA*_*t*_*, and the probability of committing type I error is not more than 0.05. Readers interested in the basic knowledge of the ET-based measurement invariance analysis can refer to Yuan and Chan (2016). In addition, we used prosocial tendencies and aggression as external criteria to evaluate their correlations with the IRI variables of the optimal model to further verify the rationality of the IRI scoring.

Descriptive statistics, alpha coefficients, and correlation analysis were performed with SPSS 23. Measurement invariance analysis was performed with R package equaltestMI (Jiang et al., 2017). All other analyses were conducted with Mplus 7.4. All the confirmatory factor analyses were performed using maximum likelihood (ML) estimation.

## Results

The descriptive statistics, the alpha coefficients of each dimension, and the correlations between the dimensions of the IRI are presented in Table 1. The results of the confirmatory factor analysis (see Table 2) show that most models did not fit well. Only the M_4c_ and M_6c_ met the empirical critical value of all fit indexes. Among the two selected models, M_4c_ is the best because almost all the fit indexes of this model are better than that of M_6c_, especially for AIC and BIC.

**TABLE 1:** Descriptive statistics, the alpha coefficients, and interdimensional correlation of the IRI.

	*M*	*SD*	1	2	3	4	5
1. PT	3.59	0.49	0.73				
2. EC	3.52	0.46	0.31***	0.68			
3. FS	3.52	0.53	0.32***	0.31***	0.68		
4. PD	3.20	0.56	0.01	0.11*	0.16***	0.73	
5. IRI	3.46	0.33	0.61***	0.68***	0.74***	0.51***	0.78

***p < 0.001; *p < 0.05. The diagonals are alpha coefficients for each dimension. PT, perspective taking; EC, empathic concern; FS, fantasy, PD, personal distress; IRI, interpersonal response index.

**TABLE 2:** Model fit indices for each IRI measurement model.

Model	*χ*^2^	*df*	*χ*^2^/*df*	CFI	NNFI	SRMR	RMSEA	RMSEA*_*t*_*	AIC	BIC
M_1a_. Four-factor model	679.569	203	3.436	0.809	0.782	**0.070**	**0.066**	**0.072**	25405.532	25713.586
M_1b_. Four-factor high-order model	700.009	205	3.415	0.801	0.776	**0.073**	**0.067**	**0.073**	25421.973	25721.469
M_1c_. Four-factor bifactor model	514.145	187	**2.749**	0.869	0.838	**0.067**	**0.057**	**0.063**	25272.109	25648.619
M_2a_. Three-factor(PT+EC+FS) model	448.910	116	3.870	0.816	0.784	**0.064**	**0.073**	0.081	19511.533	19742.573
M_2b_. Three-factor (PT+EC+FS) high-order model	448.910	116	3.870	0.816	0.784	**0.064**	**0.073**	0.081	19511.533	19742.573
M_2c_. Three-factor (PT+EC+FS) bifactor model	302.921	102	**2.970**	0.889	0.852	**0.061**	**0.061**	**0.069**	19393.544	19684.483
M_3a_. Interpersonal reactivity three-factor [empathy(PT+EC)+FS+PD] model	874.112	206	4.243	0.732	0.699	**0.079**	**0.078**	0.083	25594.076	25889.294
M_3b_. Interpersonal reactivity three-factor [empathy (PT+EC)+FS+PD] high-order model	874.112	206	4.243	0.732	0.699	**0.079**	**0.078**	0.083	25594.076	25889.294
M_3c_. Interpersonal reactivity three-factor [empathy (PT+EC)+FS+PD] bifactor model	604.125	187	3.231	0.833	0.793	**0.066**	**0.065**	**0.070**	25362.088	25738.598
M_4a_. Two-factor (PT+EC) model	142.006	43	3.302	**0.903**	0.875	**0.052**	**0.066**	**0.078**	11957.736	12103.206
M_4b_. Two-factor (PT+EC) high-order model	142.006	42	3.381	**0.902**	0.871	**0.052**	**0.067**	**0.079**	11959.736	12109.485
M_4c_. Two-factor (PT+EC) bifactor model	73.047	33	**2.214**	**0.961**	**0.934**	**0.031**	**0.048**	**0.063**	11908.777	12097.032
M_5a_. Two-factor (affective empathy: EC+PD; cognitive empathy: PT+FS) model	1313.585	208	6.315	0.557	0.507	0.107	0.100	0.105	26029.549	26316.210
M_5b_. Two-factor (affective empathy: EC+PD; cognitive empathy: PT+FS) high-order model	1313.585	207	6.346	0.556	0.505	0.107	0.100	0.105	26031.549	26322.488
M_5c_. Two-factor (affective empathy: EC+PD; cognitive empathy: PT+FS) bifactor model	780.843	187	4.176	0.762	0.706	0.081	**0.077**	0.083	25538.806	25915.316
M_6a_. Two-factor (affective empathy: EC+PD; cognitive empathy: PT) model	666.163	103	6.468	0.651	0.594	0.110	0.101	0.109	18260.189	18469.837
M_6b_. Two-factor (affective empathy: EC+PD; cognitive empathy: PT) high-order model	666.163	102	6.531	0.650	0.589	0.110	0.102	0.109	18262.189	18476.115
M_6c_. Two-factor (affective empathy: EC+PD; cognitive empathy: PT) bifactor model	193.021	88	**2.193**	**0.935**	**0.911**	**0.041**	**0.047**	**0.056**	17817.048	18090.873

*PT, perspective taking; EC, empathic concern; FS, fantasy, PD, personal distress; boldface font indicates that the fit index reaches the empirical critical value. All of the model’s *χ*^2^ estimates are significant.*

First, we focused on the two-factor (affective empathy: EC, cognitive empathy: PT) model’s corresponding bifactor model (M_4c_). In this model, the items’ loadings on the general factor were between 0.20 and 0.60 (*M* = 0.39; see Table 3), and they were higher than the empirical critical value of 0.30, indicating that there was a clear general factor. At the same time, beyond the general factor, loadings on the target local factors of PT and EC were 0.41–0.60 (*M* = 0.48) and 0.09–0.55 (*M* = 0.33), respectively, which suggested that PT and EC had a clear bifactor structure. The I-ECVs of items 1, 7, and 16 of M_4c_ were higher than 0.80, indicating that these items contribute more to the general factor than other items. Furthermore, to understand and clarify the meaning of the total score and the subscale scores, the model-based reliability indexes were calculated for this model (see Table 4). The *ω_*H*_* of this model was 0.51, which is larger than the empirical critical value of 0.50 (Reise et al., 2013), and it indicates that the total score of the PT and EC dimensions is meaningful. The *ω_*S*_* of the PT and EC dimensions were 0.74 and 0.71, respectively, and their *ω_*HS*_* were 0.48 and 0.30, respectively, and their *ω_*HS*_*/*ω_*S*_* were 0.68 and 0.42, respectively. Thus, the PT dimension’s *ω_*S*_* and *ω_*HS*_*/*ω_*S*_* reached the empirical critical value (>0.50; Reise et al., 2013), indicating that it is meaningful to score the PT dimension alone. However, the EC dimension’s *ω_*HS*_*/*ω_*S*_* was low, which means that EC did not provide much unique information after controlling for the variance due to the general factor.

**TABLE 3:** Factor loadings for the two-factor (PT+EC) bi-factor model (M_4c_).

Items of the IRI	General factor	PT	EC	Item ECV
6	**0.30**	**0.41**		0.35
9	**0.46**	**0.43**		0.54
15	**0.45**	**0.44**		0.41
19	**0.30**	**0.60**		0.20
22	0.28	**0.51**		0.24
1	**0.60**		0.21	**0.89**
2	**0.30**		**0.54**	0.24
7	**0.57**		0.09	**0.98**
11	0.20		**0.55**	0.11
14	0.26		**0.40**	0.29
16	**0.55**		0.17	**0.91**

*PT, perspective taking; EC, empathic concern; ECV, explained common variance; boldface font indicates the factor loadings above 0.30 or item ECVs above 0.80.*

**TABLE 4:** Model-based reliabilities for the selected models.

Model		*ω_*H*_*(95% CI)	*ω_*S*_*(95% CI)	*ω_*HS*_*(95% CI)	*ω_*HS*_*_/_*ω_*S*_*(95% CI)
M_4c_. Two-factor (PT+EC) bifactor model	Total scale	**0.51** (0.41, 0.60)			
	PT		**0.74** (0.70, 0.77)	0.48 (0.36, 0.59)	**0.65** (0.49, 0.80)
	EC		**0.71** (0.67, 0.75)	0.30 (0.11, 0.48)	0.42 (0.16, 0.68)
M_6c_. Two-factor (cognitive empathy: PT; affective empathy: EC+PD) bifactor model	Total scale	0.38(0.26, 0.51)			
	PT		**0.74** (0.70, 0.77)	0.56 (0.48, 0.64)	**0.76** (0.66, 0.86)
	EC+PD		**0.74** (0.71, 0.77)	0.45 (0.31, 0.60)	**0.61** (0.41, 0.81)

*PT, perspective taking; EC, empathic concern; boldface font indicates that the reliability estimates reach the empirical critical value; CI, confidence interval.*

Then, we tested the two-factor (affective empathy: EC+PD, cognitive empathy: PT) model’s corresponding bifactor model (M_6c_). In this model, items’ loadings on the general factor were between −0.16 and 0.62 (*M* = 0.28; see Table 5), and they were lower than 0.30, which indicates that the general factor is not defined clearly. Meanwhile, the loadings on the target local factors of PT and affective empathy (EC+PD) were 0.47–0.60 (*M* = 0.52) and −0.03–0.73 (*M* = 0.32), respectively. It seems that the local factor structure of this model is well defined. Nevertheless, if we carefully examine the local factor of affective empathy, we find that the loadings of the items belonging to the EC dimension originally were between −0.03 and 0.24 (*M* = 0.09) in this sample, and no item’s loading was higher than 0.30 while all target loadings on the local factors of the PD dimension were between 0.47 and 0.73 (*M* = 0.60), which was generally high. This indicates that the structure of the affective empathy factor, which was composed of EC and PD, is not ideal, and local factors reflect the connotation of PD more than EC. The contribution of EC to the general factor far outweighed the contribution to the local factor because only EC’s I-ECV is greater than 0.80. The *ω_*H*_* of this model was 0.38 (see Table 4), which is lower than 0.50, indicating that the total score of this model is statistically unmeaningful. The *ω_*S*_* of the PT and affective empathy (EC+PD) dimensions were both 0.74, and their *ω_*HS*_* were 0.56 and 0.45, respectively, and their *ω_*HS*_*/*ω_*S*_* were 0.76 and 0.61, respectively. Thus, the PT and affective empathy (EC+PD) dimensions’ *ω_*S*_* and *ω_*HS*_*/*ω_*S*_* reached the empirical critical value (>0.50), indicating that it is meaningful to use the scores of the PT and affective empathy (EC+PD) dimensions.

**TABLE 5:** Factor loadings for the two-factor (affective empathy: EC+PD; cognitive empathy: PT) bi-factor model (M_6c_).

Items of the IRI	General factor	PT	EC+PD	Item ECV
6	0.21	**0.47**		0.16
9	**0.40**	**0.49**		0.39
15	**0.41**	**0.49**		0.42
19	0.24	**0.62**		0.14
22	0.23	**0.53**		0.15
1	**0.62**		0.17	**0.93**
2	**0.53**		−0.03	**1.00**
7	**0.51**		0.16	**0.91**
11	**0.42**		−0.02	**1.00**
14	**0.43**		<0.01	**1.00**
16	**0.53**		0.24	**0.83**
4	0.12		**0.62**	0.04
8	0.12		**0.58**	0.04
13	0.07		**0.73**	0.01
18	−0.16		**0.62**	0.06
21	−0.14		**0.47**	0.08

*PT, perspective taking; EC, empathic concern, including items 1, 2, 7, 11, 14, 16; PD, personal distress including items 4, 8, 13, 18, 21; ECV, explained common variance; boldface font indicates the factor loadings above 0.30 or item ECVs above 0.80.*

Next, we tested the measurement invariance across gender of the optimal model (M_4c_). Based on ET, the fit indexes for male and female groups are both acceptable with RMSEA*_*t*_* = 0.080 and 0.057. Configural invariance is, thus, established. Then we tested metric invariance for the two groups. The RMSEA*_*t*_* for metric invariance was 0.076, indicating that the hypothesis of metric invariance was tenable. Subsequently, scalar measurement invariance in which both the items’ intercepts and factor loadings were constrained to be equal across groups was tested. The resulting RMSEA*_*t*_* was 0.048, indicating that scalar invariance was supported. Finally, based on scalar measurement invariance, latent means invariance was tested by restricting the two groups of latent means to be equal. The resulting RMSEA*_*t*_* was 0.116, indicating that latent means may not be regarded as equal across the two groups unless we can tolerate a poor model with RMSEA*_*t*_* = 0.116. By comparing the latent mean differences across gender, we found that females scored higher than males in EC (0.59, *p* < 0.001) but not PT (0.10, *p* = 0.45). Hence, overall, the results support configural, metric, and scalar invariance of M_4c_ across gender. The type I error for each step is controlled under 0.05.

In order to further verify the rationality of the use of the PT dimension score and total empathy score in the optimal model, we also calculated the partial correlations among PT dimension score, total empathy score, and prosocial behavior and aggression with demographic variables controlled. The result showed that both general empathy and PT were positively associated with prosocial tendencies and were negatively associated with aggression (see Table 6).

**TABLE 6:** Partial correlations among empathy (EC+PT), PT, and the criterion measures.

	Empathy (EC+PT)	PT	Prosocial tendencies	Aggression
Empathy (EC+PT)	1			
PT	0.77***	1		
Prosocial tendencies	0.42***	0.24***	1	
Aggression	−0.17***	−0.15***	-0.11**	1

***p < 0.001; **p < 0.01; PT, perspective taking; EC, empathic concern.

## Discussion

This study summarizes various scoring approaches of the IRI in application research and attempts to verify the rationality of these ways of scoring, especially for some scoring approaches with a high frequency of use that have not been tested (e.g., PT as the cognitive empathy, EC as the affective empathy, and their total score as empathy). The analysis results of these scoring approaches and their corresponding models are discussed below.

First, most of the IRI measurement models examined in our study do not fit well. This result is consistent with many studies of the IRI in recent years (e.g., Siu and Shek, 2005; Garcia-Barrera et al., 2017; Lucas-Molina et al., 2017; Murphy et al., 2020), indicating that the scorings based on these measurement models are statistically inappropriate. At the same time, this result, to some extent, confirms the concern of some researchers on the measurement property of the IRI (e.g., Vossen et al., 2015; Wang et al., 2017).

The bifactor model with EC and PD as affective empathy and PT as cognitive empathy (M_6c_) has an acceptable fit, and both dimension scores are meaningful according to *ω_*S*_* and *ω_*HS*_*/*ω_*S*_*. However, based on factor loadings and I-ECV, the contribution of EC is rather low on the affective empathy factor while high on the general factor. It seems that using only PD is better than using EC and PD combined as the local factor. Moreover, according to *ω_*H*_*, using the total score of this model to represent empathy is statistically inappropriate.

Relatively speaking, the bifactor model with EC as affective empathy and PT as cognitive empathy (M_4c_) has the best performance in this study. Both model fit indexes and factor loadings have reached acceptable levels. In addition, through model-based reliability, we further clarify the reasonable scoring of the IRI. Due to the high enough *ω_*H*_*, the total score of PT and EC is statistically reasonable. Additionally, according to the analysis results of the *ω_*S*_* and *ω_*HS*_*/*ω_*S*_*, it is meaningful to report the PT score alone, but the EC score should not be used separately. Based on the existing empirical studies and theoretical perspectives, we believe that there are at least two reasons for the EC dimension’s problem. First, although EC is generally regarded as a unidimensional construct (e.g., Carlo et al., 2010; Foell et al., 2018) and its connotation is very close to sympathy, in recent years, some researchers have noted that EC itself is a multidimensional construct that contains at least two psychological structures: sympathy and tenderness (Lishner et al., 2011). The difference between the two is that sympathy emphasizes the situation, it is aimed at the specific plight of others, and the sympathizers hope to provide help for the people in need at present (e.g., the item “When I see someone being taken advantage of, I feel kind of protective toward them” in EC of the IRI). On the other hand, tenderness emphasizes the object; it is targeted at the group perceived as vulnerable (such as children and small animals). Tenderness is more manifested as a kind of care for the vulnerable regardless of the specific situation (López-Pérez et al., 2017; e.g., the item “I often have tender, concerned feelings for people less fortunate than me” in EC of the IRI). Another possible cause of the EC dimension’s problem may be that there is a general response bias in the items used to measure affective empathy in the IRI. Chrysikou and Thompson (2016) point out that the items used to measure affective empathy in the IRI require individuals to use cognitive empathy first, thus enabling them to be in the situations created by the items before making emotional responses, which makes it difficult or impossible for these items to measure affective empathy purely.

The measurement invariance analysis across gender for the bifactor model with EC as affective empathy and PT as cognitive empathy (M_4c_) endorsed the configural, metric, and scalar invariance but not latent mean invariance. The EC score of the females was higher than the males, which was consistent with the previous study of measurement invariance for the IRI (Lucas-Molina et al., 2017). With respect to the evidence of criterion-related validity for M_4c_, both general empathy and PT were positively associated with prosocial tendencies and were negatively associated with aggression. This is in line with relevant theories on empathy and PT and further justifies the scoring of general empathy (EC+PT) and PT.

There are some limitations of the current study. First, although our confirmatory factor analysis results show that the total score of PT and EC is statistically meaningful, such an analysis does not prove that the total score is also theoretically feasible. This issue needs to be addressed through validity analysis. Our criterion-related validity analysis through prosocial tendencies and aggression proved to some extent that it is theoretically meaningful to use the sum of PT and EC to represent empathy. However, this validity analysis still has limitations. To be specific, instead of the narrow definition of empathy (that is, affective empathy means experiencing similar or even identical emotions with others, and cognitive empathy means understanding other people’s emotions only; Vachon et al., 2014), the definition of empathy in the IRI is based on a broad definition; it regards factors such as sympathy and understanding of the thoughts of others as components of empathy as well. Although some researchers support this definition of empathy (e.g., Vachon et al., 2014), other researchers think that this definition may blur the boundaries of empathy and lead to incorrect research conclusions and misunderstanding of the research results of others (e.g., Vossen et al., 2015). Considering that both narrowly and broadly defined empathy is thought to be positively correlated with prosocial tendencies and negatively correlated with aggression, the scoring of the IRI suggested in this study should also be used with great caution, and researchers need to clarify the definition of empathy they use to properly interpret the research conclusions.

Second, the conclusions of this study are based on a student sample with the IRI-C, which cannot guarantee that the results can be generalized to all subject groups and all four-factor versions of the IRI. Therefore, it is necessary to use more samples and more four-dimensional versions of the IRI to verify the replicability and correctness of this study. Nevertheless, this study still verifies some previous research results (e.g., Siu and Shek, 2005; Chrysikou and Thompson, 2016; Murphy et al., 2020); it can increase our confidence in the conclusions of some IRI scoring approaches’ irrationality and can give researchers a caution against using these scorings. Additionally, this article shows researchers who want to use the IRI score flexibly how to judge whether certain scoring methods are appropriate.

Third, social desirability was not examined in the present research due to the length of the questionnaire. Considering the measurement of empathy may be susceptive to social desirability (Miklikowska, 2018), psychometric research of the IRI in the future should better include social desirability as a covariate.

Another limitation of this study is that the evaluation of models is based on the specific cutoff points given that the selection of different cutoff points may change the results of the study. For example, if 0.05 and 0.95 were selected as the acceptable levels for RMSEA and NNFI, respectively, all the models in this study would be rejected.

Finally, in this study, confirmatory factor analysis adopts ML, which requires data to be continuous, but the data for a Likert scale are ordered categorically in nature. Although ML is somewhat robust for categorical data under certain conditions (e.g., skewness < 2 and kurtosis < 7 with scale points no less than five categories; see Curran et al., 1996; Rhemtulla et al., 2012; Boateng et al., 2018), we still hope that future studies replicate the results of current study using the methods designed to deal with categorical data, such as weighted least squares means and variance adjusted estimation (WLSMV).

## Conclusion

In this study, the IRI was used to conduct a confirmatory factor analysis, and the results show that several common approaches of scoring the IRI in empirical studies are unreasonable, such as adding the PT, EC, and FS dimensions to represent empathy or adding the PT and FS dimensions as cognitive empathy and adding the EC and PD dimensions as affective empathy. As a result, the credibility of the conclusions of relevant studies based on these IRI scoring approaches is also questionable. We suggest that the IRI scores should be used more carefully in future studies. A better scoring approach is to add the PT and EC dimension as the total score of the IRI and to report the PT score separately as the measure of cognitive empathy.

## Data Availability Statement

The datasets generated for this study are available on request to the corresponding author.

## Ethics Statement

The studies involving human participants were reviewed and approved by the Ethics Committee of the School of Public Administration, Guangdong University of Finance. Written informed consent to participate in this study was provided by the participants.

## Author Contributions

YW contributed to project design and administration, survey creation, data collection, data preparation and coding, data analysis, and manuscript writing. YL contributed to manuscript writing, comment, and revision. WX contributed to project administration, survey creation, data collection, data preparation, and coding. YF contributed to manuscript comment and revision and data interpretation. JJ contributed to manuscript comment and revision.

## Conflict of Interest

The authors declare that the research was conducted in the absence of any commercial or financial relationships that could be construed as a potential conflict of interest.
